# Comparative Echocardiographic Evaluation of Right Pulmonary Artery Dimensions and Right Pulmonary Artery Distensibility Index in Dogs with Heartworm Disease

**DOI:** 10.3390/vetsci12030246

**Published:** 2025-03-04

**Authors:** Jorge Isidoro Matos, Alicia Caro-Vadillo, Eva Mohr-Peraza, Sara Nieves García-Rodríguez, José Alberto Montoya-Alonso, Elena Carretón

**Affiliations:** 1 Internal Medicine, Veterinary Medicine and Therapeutic Research Group, Faculty of Veterinary Science, Research Institute of Biomedical and Health Sciences (IUIBS), University of Las Palmas de Gran Canaria, 35017 Las Palmas de Gran Canaria, Spain; jorge.matos@ulpgc.es (J.I.M.); eva.mohr@ulpgc.es (E.M.-P.); alberto.montoya@ulpgc.es (J.A.M.-A.); elena.carreton@ulpgc.es (E.C.); 2 Department of Animal Medicine and Surgery, Faculty of Veterinary Medicine, Complutense University, 28040 Madrid, Spain; aliciac@vet.ucm.es

**Keywords:** veterinary, cardiology, pulmonary hypertension, echocardiography, pulmonary artery, RPADi, heartworm, *Dirofilaria immitis*

## Abstract

The existence of several methods to obtain the right pulmonary artery distensibility index (RPADi) has complicated the standardization and use of echocardiographic measurement for the determination of pulmonary hypertension (PH) in heartworm disease and other cardiorespiratory pathologies. Therefore, the objective was to determine the precision and agreement between RPADVisser and RPADVenco in the diagnosis of PH and to evaluate the differences in pulmonary arterial diameters in dogs with heartworm. The RPADVisser and RPADVenco were determined in 248 dogs and the determinations of RPADmax, RPADmin, RPADmax:Ao, and RPADmim:Ao were also obtained from each of the methods. Through the presence/absence of heartworm disease and the presence of PH (RPADi < 30% in both methods), three groups were created: healthy animals, infected dogs without PH, and animals infected with PH. Statistical analyzes determined significant differences between the analyzed groups for RPADmax, RPADmin, RPADmax:Ao, and RPADmim:Ao. Likewise, a high similarity and agreement was reported between RPADVisser and RPADVenco; however, 5.6% of the cases showed a different diagnosis for the presence of PH in the animals analyzed, showing that these methods cannot be used interchangeably.

## Simple Summary

The existence of two methods for obtaining the right pulmonary artery distensibility index (RPADi) has complicated the standardization and use of echocardiographic measurements in determining pulmonary hypertension (PH) in heartworm disease and other cardiorespiratory conditions. This study aimed to determine the accuracy and agreement between RPADVisser and RPADVenco in diagnosing PH and to assess the differences in pulmonary arterial diameters in dogs with heartworm. RPADVisser and RPADVenco were measured in 248 dogs, along with the values for RPADmax, RPADmin, RPADmax:Ao, and RPADmin:Ao for each method. The dogs were grouped into three categories: healthy, infected without PH, and infected with PH. Statistical analyses revealed significant differences between these groups for RPADmax, RPADmin, RPADmax:Ao, and RPADmin:Ao. Additionally, this study found high similarity and agreement between RPADVisser and RPADVenco. However, 5.6% of the cases showed differences in the diagnosis for PH, indicating that these methods cannot be used interchangeably.

## 1. Introduction

Canine cardiopulmonary dirofilariosis or heartworm disease (*Dirofilaria immitis*), is a globally spread vector-borne disease. In dogs, the presence of adult parasites causes endothelial damage in the pulmonary arteries, leading to the appearance of proliferative pulmonary endarteritis. The chronicity of endothelial lesions and the perpetuation of adult parasites trigger phenomena of pulmonary hypertension (PH) of precapillary origin [1,2,3].

The presence of PH is a common and frequent sign in canine heartworm, affecting around 30–60% of the parasitized animals [4,5], causing cardiorespiratory symptoms and right congestive heart failure. The clinical importance in endemic areas and in those where the disease is expanding make the diagnosis of PH produced by *D. immitis* of mandatory interest [3,4,5].

The right pulmonary artery distensibility index (RPADi) is an echocardiographic measure used to assess the probability of suffering from PH in dogs. Traditionally, the echocardiographic measurement of the RPADi, has proven to be useful for the diagnosis and staging of PH in heartworm disease, especially when there is no possibility of measuring tricuspid or pulmonary regurgitation flows [6,7,8,9]. Likewise, it has been shown to be an adequate index in the study of other cardiorespiratory pathologies that generate PH, in which, as in heartworm disease, dilation of the right pulmonary artery occurs, in addition to a loss of compliance during cardiac systole and diastole [9,10,11,12]. The cut-off point of <30% has been widely used in previous studies involving the diagnosis of moderate or severe PH (>50 mmHg), both in heartworm disease and in other pathologies [5,8,11,12,13,14,15]. However, the existence of two performance methods to obtain the RPADi complicates the standardization and use of the echocardiographic measurement at present. On the one hand, the use of study level through a long axis right parasternal view using monodimensional mode (M-Mode) developed in veterinary medicine following the indications of Venco et al. [8], and on the other hand the use of a short axis right parasternal view at of the bifurcation of the branches of the pulmonary trunk using a bidimensional mode (2D mode) developed by Visser et al. [11].

Recently, a study assessed the differences between these methods for determining the RPADi, reporting differences between them and concluding that cannot be used interchangeably in the detection of PH [10]. However, a larger sample size and other echocardiographic variables involved in the determination of RPADi in dogs with heartworm must be evaluated to confirm the previous findings. Therefore, the aim of the present research was to ascertain the precision and concordance between both methods of RPADi determination in the diagnosis of PH in canine heartworm disease.

## 2. Materials and Methods

**Animals**

The study subjects consisted of 248 privately owned dogs attending at the Veterinary Teaching Hospital of the University of Las Palmas de Gran Canaria (Canary Islands, Spain). All dogs lived in a hyperendemic area and without prophylactic measures against heartworm infection [16] and attended a heartworm screening campaign. A complete record of each animal was kept, including age, sex, breed, weight, presence of cardiorespiratory symptoms associated with heartworm disease (dyspnea, cough, exercise intolerance, weakness, wight loss, and syncope), and also signs of right-sided congestive heart failure (R-CHF), mainly based on evidence of ascites, pleural effusion, jugular pulse and cava vein distention. Animals receiving any medication for previous cardiorespiratory diseases were excluded from this study.

Of the included dogs, 71.37% (177/248) were diagnosed with heartworm disease using a commercial immunochromatographic test kit (Urano test Dirofilaria, Urano Vet SL, Barcelona, Spain), while 28.63% (71/248) were considered healthy based on the absence of clinical signs, history, physical examination, thoracic radiographs, cardiovascular and echocardiographic evaluation, and negative *D. immitis* antigen detection test results. Dogs with evidence of cardiac or respiratory conditions coexisting with heartworm disease were excluded from this study.

**Echocardiography**

All dogs included in this study underwent echocardiographic examination, which were performed between September 2021 and October 2023 by the same veterinary cardiologist using a Vivid Iq ultrasonographer (General Electrics, Boston, MA, USA) equipped with 2.5–12.5 MHz phased array transducers. No dogs were sedated or anesthetized, and an electrocardiographic monitoring was maintained throughout the examination. The average measurement of three consecutive cardiac cycles in sinus rhythm was determined to avoid any influence of the respiratory and cardiac cycles.

**Figure 1:**
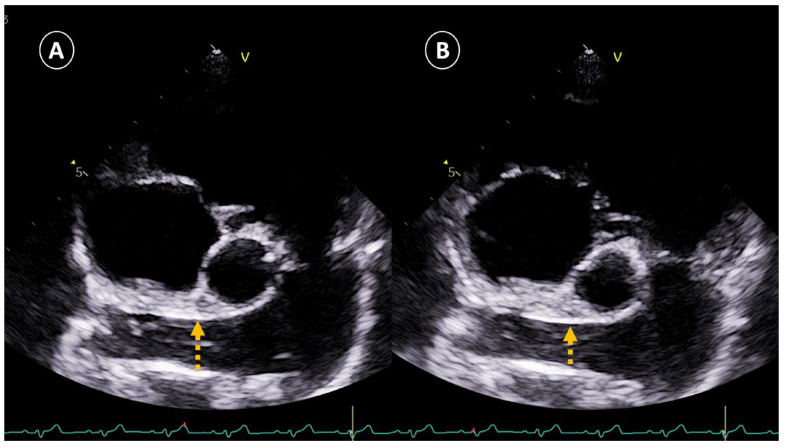
Representative image of the right pulmonary artery distensibility index (RPADi) measurement using the method of Visser et al. [10] in a dog with heartworms and pulmonary hypertension. The right parasternal short-axis view was used at the level of the pulmonary trunk bifurcation. An adequate two-dimensional image with direct visualization of the longitudinal axis of the right pulmonary artery was obtained. Systolic diameter (**A**) was measured at the maximum dimension (T wave), and diastolic diameter (**B**) at the smallest dimension (Q wave). Internal diameters were measured with the “trailing edge to leading edge” method, using the same location of the right pulmonary artery.

**Figure 2:**
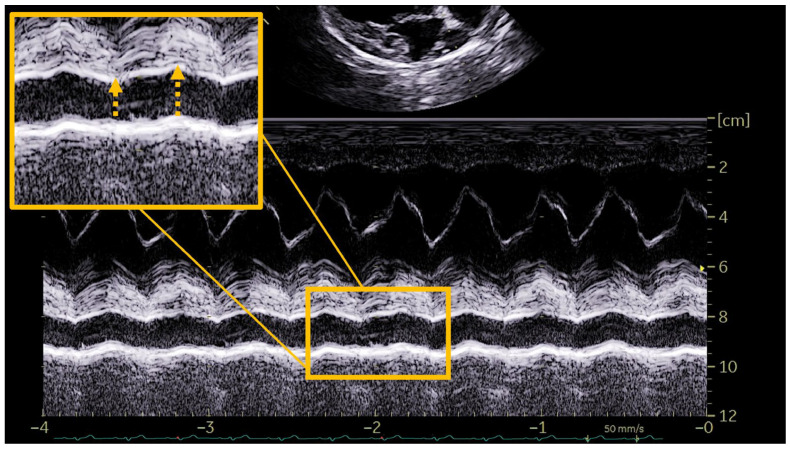
Representative image of the right pulmonary artery distensibility index (RPADi) measurement using the method of Venco et al. [6] in a dog infected by *Dirofilaria immitis* and with pulmonary hypertension. The right parasternal long-axis view of four cardiac chambers was optimized and M-mode was used on the transverse axis of the right pulmonary artery when the left atrium and pulmonary artery and vein were accurately observed. Systolic diameter was measured at the maximum dimension (T wave) and diastolic diameter at the smallest dimension (Q wave). The right pulmonary artery diameters were measured using the “leading edge to leading edge” measurement convention.

The determination of the presence of PH was based on the two different methods of obtaining the RPADi through the use of echocardiographic M-mode and 2D mode, being a value < 30% correlated in both cases with the presence of moderate or severe PH (>50 mmHg) [8,11]. To determine the RPADi using the Visser et al., method [11], the right parasternal short-axis view was applied at the level of the pulmonary trunk bifurcation, clearly observing the progression of the right and left branches. An adequate two-dimensional image with direct visualization of the longitudinal axis of the right pulmonary artery was obtained in all cases. The minimum diastolic and maximum systolic internal diameters were measured with the “trailing edge to leading edge” (te-le) method, using the same location of the right pulmonary artery (RPADVisser). Special care was taken to take measurements of the farthest portion, avoiding including the aorta (Ao) in the image (Figure 1). On the other hand, the determination of the RPADi using the Venco et al. method [8] was based on the right parasternal long-axis view of four cardiac chambers. M-mode was used in this case on the transverse axis of the right pulmonary artery when the left atrium, and pulmonary artery and vein were accurately observed. The right pulmonary artery diameters were measured using the “leading edge to leading edge” (le-le) measurement convention (RPADVenco) (Figure 2). In both cases, systolic diameter was measured at the maximum (T wave) and diastolic diameter at the smallest (Q wave) dimensions. The RPADi was calculated as the percentage difference in right pulmonary artery diameter in systole and diastole, using the following formula: RPADi = [(RPAmax − RPAmin)/RPAmax] × 100, as previously described [10].

Additionally, in each of the methods evaluated (RPADVisser and RPADVenco) the maximum (RPAmax) and minimum (RPAmin) diameters of the right pulmonary artery were described and compared. Likewise, the RPAmax and RPAmin of each of the methods were indexed to the diameter of the Ao measured from a right parasternal long-axis view and optimized for the left ventricular outflow tract. Measurement of the Ao was performed during early systole at the base of the aortic leaflets, visible at maximum open aortic valve. In this way, the RPAmax:Ao and RPAmin:Ao ratios were obtained for each of the RPADi methods [17].

Finally, the estimated burden of adult worms (I–IV) was classified according to Venco et al. [18]. Low parasite burden (I–II) was determined when no parasites were echocardiographically observed or when they were observed only in the right pulmonary branch, and a high parasite burden (III–IV) was determined when adult worms were observed in the pulmonary trunk or when adult parasites were observed in the right heart chambers.

**Statistical analysis**

For categorical variables, frequencies, and percentages were shown. For continuous variables, the descriptive of the mean, standard deviation, median, and interquartile range were shown. The differences in parameters between groups were evaluated, if continuous, using Mann–Whitney (non-parametric) or T-student (parametric) tests based on the normality of the variables to be evaluated using the Shapiro–Wilks test and, if the variables were categorical, using Pearson’s Chi^2^ test. All multiple comparisons were adjusted by Bonferroni correction. All contrasts were accompanied by the effect size estimator to complete the interpretation of the results: Cramer’s V for categorical variables and Cohen’s d for continuous variables. The criterion for Cohen’s d for classifying the magnitude of the effect was as follows: small (d = 0.2–0.4), medium (d = 0.5–0.8), and large (d = greater than 0.8); For Cramer’s V it was the following: 0.00–0.09 as negligible, 0.10–0.29 as low, 0.30–0.49 as medium and from 0.50 as high. For agreement between methods, the Pearson correlation and the Intraclass Correlation Coefficient (ICC) were applied, which allowed measuring the agreement between two or more quantitative assessments obtained with different measuring instruments or evaluators. In addition, the McNemar test compared the percentages of change established by both methods (PH with one method and non-PH with the other and vice versa). To complement the agreement between methods, the ROC curve was calculated for each method with respect to the Yes/No PH classification obtained by the other method. Choosing the value of the parameter that maximizes the Youden index (sensitivity + specificity − 1) as the cut-off point, the best possible approximation to the real classification was obtained. Finally, the Kappa concordance index was used to evaluate the degree of concordance between the results provided by both observers. The Kappa index is a parameter whose maximum possible value is 1 (total concordance). The level of significance used in the analysis was 5% (α = 0.05).

**Ethical statement**

All owners were informed and gave their consent to participate in this study. Ethical approval was not required for this study, as it was a purely observational study with voluntary enrollment and did not involve additional invasive clinical diagnostic procedures. The evaluation of this study included ethical considerations and legal aspects regarding animal protection and welfare and was carried out in accordance with the current Spanish and European legislation on animal protection.

## 3. Results

All controls were healthy without presence of PH and, among the patients with heartworm, 43.5% had PH based on RPADVisser and 48% based on RPADVenco. According to the results, dogs were divided into three groups: Group A (*n* = 71) included healthy animals, Group B (*n* = 100 for RPADVisser and *n* = 92 for RPADVenco) were heartworm-infected animals without PH, and Group C (*n* = 77 for RPADVisser and *n* = 85 for RPADVenco) were dogs with heartworm and PH.

Table 1 shows the descriptive results for the three groups of dogs for each method. The groups were fairly homogeneous, with no significant differences in the results with respect to sex, race, weight or age between the groups studied. Respiratory symptoms and the presence of R-CHF were significantly related to the disease and the presence of PH in both RPADi methods. The parasite burden also increased with the PH in both methods: all dogs in Group A showed absence of parasites; 27% (RPADVisser) − 29% (RPADVenco) of dogs in Group B had high parasite burden; and 72% (RPADVisser) − 74% (RPADVenco) of dogs in Group C had high parasite burden. Moreover, the measurements of RPADmax, RPADmin, RPADmax:Ao, and RPADmim:Ao reported significant differences between RPADVisser and RPADVenco.

**Figure 3:**
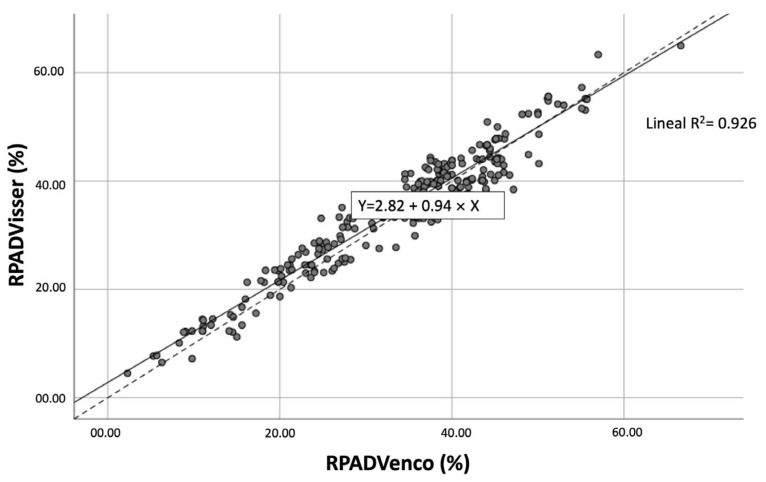
Scatter plots showing significant (*p*-value < 0.001) correlations (R^2^ = 0.926) between the RPADVisser and RPADVenco measurements. The solid line within each scatterplot represents the line of best fit. The Pearson correlation between the two ratios was very high: 0.963 (*p*-value < 0.001).

The large sample size of this study allowed assuming the normality of the two methods to be compared by applying the Central Limit Theorem. The Pearson correlation between the two ratios was very high: 0.963 (*p*-value 0.000) (Figure 3).

To assess the agreement between the methods, the intraclass correlation coefficient (ICC) was calculated. Both the ICC of absolute agreement and that of consistency were excellent with values > 0.95 [ICC absolute agreement: 0.979 (*p*-value 0.000); ICC consistency: 0.981 (*p*-value 0.000)]. However, the *p*-value of the t-student test for paired samples was 0.000 which indicated significant differences in the values of the two indices.

**Figure 4:**
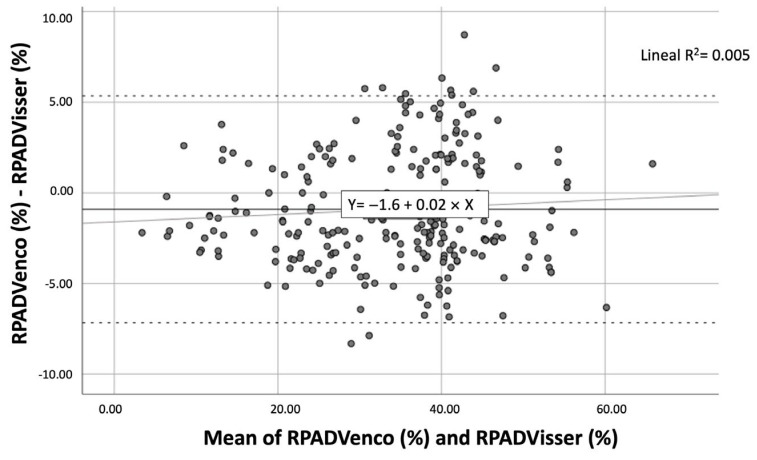
Scatter plots showing the Bland–Altman representation of the RPADVisser and RPADVenco measurements. The point cloud was distributed quite homogeneously around zero except for some values below 30 where RPADVisser scored much higher, and some above 30 where the RPADVenco scored much higher.

The Bland–Altman representation investigated how the differences between the methods behave (Figure 4). The bias (mean of the differences) between methods was −0.9069 (solid line) with a t-student *p*-value of 0.000, which meant that on average the RPADVenco and RPADVisser indices did not measure statistically equal values. Approximately 95% of the differences between methods were between −7.1673 (−0.9069 − 1.96 × SD) and 5.3535 (−0.9069 + 1.96 × SD)—dashed lines. The regression line calculated for the differences was practically horizontal and almost overlapped with the bias line, so there was no proportional bias but a constant bias, that is, the cloud of points was distributed quite homogeneously around zero, except for some values below 30 where RPADVisser scored much higher, and some above 30 where RPADVenco scored much higher (points outside the solid lines). In other words, there was no pattern of discordance, but sometimes one index scored higher and sometimes the other, without following any order.

This comparative study, by establishing a cut-off point for RPADi < 30% [PH No (≥30%)/Yes (<30%)], allowed a comparison the classification of dogs by using both methods: 1.8% (*n* = 3) of the dogs detected as normotensive by RPADVenco were classified as dogs with PH by RPADVisser and, on the contrary, 13% (*n* = 11) of the dogs with PH by RPADVenco were classified as normotensive dogs by RPADVisser. On the other hand, 6.4% (*n* = 11) of the normo-tensive dogs detected by RPADVisser were classified as dogs with PH by RPADVenco and, conversely, 2.9% (*n* = 3) of the dogs with PH by RPADVisser were normotensive dogs by RPADVenco (Table 2).

The statistical values of both measurements were represented depending on the presence or absence of PH through the mean, standard deviation, median, 25th quartile, and 75th quartile. The values were around the cut-off point of 30% with interquartile ranges with limits between 27% and 33% (Table 3).

To verify the diagnostic capacity of both indices, two ROC CURVES were applied—one for each index—in which the events to be studied were as follows: suffering from PH (RPADi < 30%) determined with RPADVisser for the RPADVenco and suffering from PH (RPADi < 30%) determined with RPADVenco for the RPADVisser (Table 4). The results showed that the areas under the curve (AUC) of the two parameters were excellent (>0.95): Any value ≤ 27.7 of RPADVisser led to PH (RPADVenco < 30%) in 94.8% of cases and a value > 27.7 led to normotension (RPADVenco ≥ 30%) in 97.1% of cases. Any value ≤ 31.83 of RPADVenco led to PH (RPADVisser < 30%) in 92.8% of cases and a value > 31.83 led to normotension (RPADVisser ≥ 30) in 96.9% of cases.

Finally, the Kappa index for the concordance of both methods in the classification of hypertension was 0.872 (*p* < 0.001), indicating very good concordance.

## 4. Discussion

The RPADi measurement is an echocardiographic determination widely used in both human and veterinary medicine in the diagnosis of PH, both of precapillary [5,6,7,8,9,12,15,19,20,21,22,23,24,25,26] and postcapillary [11,13,14,20,22,23,24] origin. In particular, it is considered a measure of choice when Doppler tracing of pulmonary or tricuspid regurgitation is not available [1,21]. The origin of PH seems to be of particular relevance to the usefulness of RPADi; in general, previous studies have found that RPADi values are lower in animals with PH affected by precapillary causes, with both RPADVenco and RPADVisser echocardiographic measurement, and a direct correlation with the measurement of tricuspid regurgitation if present [12,13,24,25,26]. This is probably due to direct damage to the pulmonary arterial vasculature in precapillary PH, versus retrograde damage from increased pressure in the pulmonary venous vasculature due to left heart pathology in postcapillary PH. 

More specifically, in the case of heartworm disease, a good correlation of RPADi with measurements made by invasive direct right heart catheterization (gold standard) has been described for the estimation of PH [8] and the use of RPADi has been the most widely used measure to determine the presence of PH in dogs parasitized by *D. immitis* [5,6,7,8,9,15], generally RPADVenco. In fact, several studies have investigated the RPADi in dogs with heartworm and other diseases using the RPADVenco, determining that the lowest values were found in animals infected with *D. immitis* [13,23,25]. Since pulmonary vascular lesions generated by the presence of adult worms cause endothelial damage, dilatation, loss of structure, tortuosity, dilatation of damaged pulmonary arteries, and PH [22], their determination by RPADi is considered to be of special interest [5,6,7,8,9].

However, in veterinary medicine there is no standardized protocol for RPADi determination [10]. There are studies performed by longitudinal right parasternal view [3,6,7,8,11,13,23,24] and others by transverse view [6,7,11,12,20,24], as well as 2D mode [5,7,11,12,20,24] or M-mode has also been used for RPADi measurement [5,8,9,13,14,15,25,26]. Variations have also been observed regarding the determination of the diameters in systole or diastole of the right pulmonary artery (trailing edge-to-leading Edge [6,7,11,12,20,24] or leading edge-to-leading Edge [5,8,9,13,15,25,26]). Therefore, it was necessary to carry out studies comparing the different methodologies described.

In the present study, RPADi measurement was investigated in 248 dogs, 71.4% of which had heartworm disease. Statistical analysis between the RPADVisser and RPADVenco methods showed that the two methods were not interchangeable with each other, although a good correlation between the methods was recorded. These results were similar to those reported by Basile et al. [10], who also evaluated in 46 healthy and heartworm-infected dogs the comparison between methods of estimating PH using the RPADVisser and RPADVenco methods, in addition to some modifications for both methods. The authors concluded that, although the Bland–Altman test showed statistical agreement between the different methods used to calculate the RPADi, the results of both methods varied, so the method of measurement used when evaluating an animal should always be specified.

On the other hand, the study developed by Vezzosi et al. [23] determined that both methods could be used interchangeably to assess RPADi, by comparing the two echocardiographic views (short axis and a long axis) to obtain the RPADi, but using M-mode in both measurements; the Pearson test showed a strong positive linear correlation between the RPADi values obtained with both methods (r^2^ = 0.9346) and the Bland–Altman test showed good agreement between the two methods in the estimation of RPADi, the mean difference between the two methods being 0.51%. This difference in results could be due to the sample size, the pathologies involved in the study—presumably causing PH of post-capillary origin—and the difference in the methodology used for RPADi measurement, due to the fact that M-mode was used in both cases.

The RPADVenco and RPADVisser methods, although measuring the same vascular structures, do so in completely different ways. The results obtained and previously published references reinforce the idea that they are useful measures but should be used independently and not interchangeably to determine the presence of PH in veterinary medicine, especially in the determination of PH in heartworm disease [10].

As in previous studies performed on heartworm disease, no relationship between the presence of PH and age, breed, weight, or sex was observed [5,8,9,15]. Also, respiratory symptoms and R-CHF were directly related to the presence of PH, as previously described [7,8,13,27]. The results showed that high parasitic burden does appear to be a predisposing factor for PH which may be explained by increased endothelial damage, as well as increased ability to impede blood flow and increased risk of thromboembolism, caused by a higher relative presence of worms [9,15]. However, in contrast to the present study, other authors observed that there were no significant differences in the presence or absence of PH between animals with low or high parasite burden, as estimated by the RPADi [5,6] or other echocardiographic parameters [27], and that the chronicity of the disease, the intensity of exercise and the immune response of the host to the parasite may exert a greater influence on the development of PH [8,28]. Nevertheless, in the conducted research, other echocardiographic determinations routinely used in the diagnosis of PH were not included, which can be considered a limitation in interpreting the obtained results. Likewise, the results obtained must be analyzed while considering the specific conditions of the patients included in this study. Factors such as the wide variation in body weight, age, and breed were not analyzed, and their impact on the results remains unknown.

To the authors’ knowledge, RPADmax:Ao and RPADmin:Ao measurements have not been previously evaluated in heartworm disease; therefore, this study described those values based on the two RPADi methods for the first time. RPADmax, RPADmin, RPADmax:Ao, and RPADmin:Ao measurements were slightly lower with the PRADVenco method compared to RPAD-Visser. These differences may be explained by the different location of the determination of the systolic and diastolic diameters of the right pulmonary artery, as well as the difference in the use of M-mode or 2D-mode and whether or not the arterial vascular wall was added to the measurement. Recently, Grosso et al. [17] showed reference values for RPADmax, RPADmin, RPADmax:Ao, and RPADmin:Ao for healthy dogs using the RPADVisser method. The results for the healthy dogs in the present study showed values within these ranges for both RPADVisser and RPADVenco.

## 5. Conclusions

The present study showed that the main methods of the RPADi determination, RPADVisser and RPADVenco, are highly similar to each other in determining the presence of PH in dogs with heartworm disease. However, slight but statistically significant differences have been found between them, which indicate that they are not interchangeable and must be used independently of each other. Similarly, different values were reported for RPADmax, RPADmin, RPADmax:Ao, and RPADmim:Ao between the methods analyzed, highlighting the differences between the technique used to determine the RPADi.

## Figures and Tables

**Table 1:** Epidemiological, clinical, and echocardiographic parameters of the dogs studied (*n* = 248). Data represent median and standard deviation unless otherwise indicated. Group A: healthy dogs; Group B: dogs with heartworms and absence of pulmonary hypertension (PH); Group C: dogs with heartworms and with PH. Legend: RPADVisser: right pulmonary artery distensibility index measured following the Visser et al. method [10]; RPADVenco: right pulmonary artery distensibility index measured following the Venco et al. method [6]; R-CHF: right congestive heart failure; HR: heart rate; RPADmim: minimum diameter of the right pulmonary artery; RPADmax: maximum diameter of the right pulmonary artery; Ao: Aorta. Results for female, mongrel, and R-CHF, high parasite burden and respiratory symptoms are expressed as *n* (%). Significant differences were considered when the *p* value < 0.05.

	RPADVisser (*n* = 248)				RPADVenco (*n* = 248)			
Parameter	Group A (*n* = 71)	Group B (*n* = 100)	Group C (*n* = 77)	*p*-Value (Cohen’s d or Cramer’s V Value)	Group A (*n* = 71)	Group B (*n* = 92)	Group C (*n* = 85)	*p*-Value (Cohen’s d or Cramer’s V Value)
Age (years)	8.32 ± 3.78	8.24 ± 3.78	8.07 ± 3.78	0.176	8.32 ± 3.78	8.08 ± 2.94	8.06 ± 3.58	0.234
Body weight (kg)	16.60 ± 10.17	16.60 ± 10.17	16.22 ± 10.29	0.106	16.60 ± 10.17	16.56 ± 9.72	16.31 ± 10.20	0.232
Female: number (%)	39 (54.9%)	56 (56%)	39 (50.6%)	0.222	39 (54.9%)	51 (55.4%)	42 (49.4%)	0.199
Mongrel: number (%)	21 (29.6%)	51 (51%)	40 (51.9%)	0.268	21 (29.6%)	48 (52.2%)	43 (50.6%)	0.119
Respiratory symptoms: number (%)	0 (0%)	35 (35%)	64(83.1%)	0.000 (0.66)	0 (0%)	28 (30.4%)	71 (83.5%)	0.000 (0.69)
R-CHF: number (%)	0 (0%)	0 (0%)	30 (39%)	0.000 (0.54)	0 (0%)	0 (0%)	30 (35.3%)	0.000 (0.50)
High parasite burden: number (%)	0 (0%)	29 (29%)	57 (74%)	0.000 (1.23)	0 (0%)	25 (27.2%)	61 (71.8%)	0.000 (1.16)
RPADmin (mm)	5.07 ± 1.82	5.14 ± 1.94	11.64 ± 1.84	0.000 (1.27)	3.77 ± 1.37	3.86 ± 1.43	10.14 ± 1.46	0.000 (1.56)
RPADmax (mm)	8.64 ± 3.15	8.86 ± 3.22	15.07 ± 3.19	0.000 (1.31)	7.11 ± 2.36	7.02 ± 2.31	13.71 ± 2.41	0.000 (1.22)
RPADmin:Ao	0.41 ± 0.22	0.38 ± 0.27	0.69 ± 0.24	0.000 (1.12)	0.35 ± 0.31	0.30 ± 0.38	0.61 ± 0.36	0.000 (1.13)
RPADmax:Ao	0.70 ± 0.16	0.67 ± 0.21	0.91 ± 0.18	0.000 (1.05)	0.51 ± 0.22	0.54 ± 0.27	0.82 ± 0.25	0.000 (1.10)

*p* < 0.01 = ANOVA and Cohen’s d value in brackets; *p* < 0.01 = Mann–Whitney/Kruskall–Wallis and Cohen’s d value in brackets; *p* < 0.01 = Chi^2^ test and Cramer’s V in brackets.

**Table 2:** Discordant pulmonary hypertension (PH) diagnosis groups between the RPADVisser and RPADVenco measurements in the studied dogs (*n* = 248).

			RPADVenco (%)		Total
			No PH	Yes PH	
RPADVisser (%)	No PH	Count	160	11	171
		% of total	64.5%	4.4%	69.0%
	Yes PH	Count	3	74	77
		% of total	1.2%	29.8%	31.0%
Total		Count	163	85	248
		% of total	65.7%	34.3%	100.0%

**Table 3:** Descriptive statistical values between the RPADVisser and RPADVenco methods in the dogs studied (*n* = 248) depending on the presence or absence of pulmonary hypertension (PH).

		Presence PH RPADVenco (<30%)			
		No		Yes	
		Presence PH RPADVisser		Presence PH RPADVisser	
		No	Yes	No	Yes
RPADVenco (%)	Valid N	160	3	11	74
	Mean	41.15	31.68	27.60	19.02
	Standard deviation	6.04	1.75	1.20	6.45
	Median	40.00	31.55	27.80	20.30
	25th percentile	37.01	30.00	27.00	14.51
	75th percentile	44.47	33.50	28.50	24.44
RPADVisser (%)	Valid N	160	3	11	74
	Mean	41.66	27.80	32.60	20.37
	Standard deviation	6.39	0.28	1.51	6.56
	Median	40.28	27.75	33.10	23.05
	25th percentile	37.60	27.55	31.44	14.50
	75th percentile	44.08	28.11	33.33	25.50

**Table 4:** Results of area under the curve (AUC), confidence interval 95% (CI95%), sensitivity (Se), specificity (Sp), and Youden index of cut-off points of RPADVisser y RPADVenco measurements for predicting pulmonary hypertension (PH) for detecting RPADiVenco and RPADVisser <30%, respectively.

Method	Event	AUC	CI 95%	Cut-Off	Sen	Sp	YOUDEN Index (Sen + Sp − 1)
RPADVisser (%)	RPADVenco < 30%	0.989	(0.981, 1.000)	≤27.70	0.948	0.971	0.919
RPADVenco (%)	RPADVisser < 30%	0.994	(0.988, 1.000)	≤31.83	0.918	0.969	0.887

## Data Availability

All data generated or analyzed during this study are included in this article. The datasets used and/or analyzed during the present study are available from the corresponding author upon reasonable request.
